# Supplementary material for: Ethanol Coupling Reactions over MgO–Al2O3 Mixed Oxide-Based Catalysts for Producing Biofuel Additives
Source: Molecules. 2023 Apr 28;28(9):3788. doi: 10.3390/molecules28093788 (PMC10180391; doi:10.3390/molecules28093788)
Supplement: Supplementary file 1 [file molecules-28-03788-s001.zip › molecules-2340650-supplementary.pdf]

Supplementary Materials

Ethanol Coupling Reactions over MgO–Al_2O_3 Mixed Oxide-Based Catalysts for Producing Biofuel Additives

Anna Vikár [1], Ferenc Lónyi [1,*], Amosi Makoye [1,2], Tibor Nagy [1], Gyula Novodárszki [1], Róbert Barthos [1], Blanka Szabó [1], József Valyon [1], Magdolna R. Mihályi [1], Dhanapati Deka [3] and Hanna E. Solt [1]

1 Institute of Materials and Environmental Chemistry, Research Centre for Natural Sciences, Magyar Tudósok Körútja 2, 1117 Budapest, Hungary.
2 Hevesy György Doctoral School of Chemistry, ELTE Eötvös Loránd University, Pázmány Péter s. 1/A, 1117 Budapest, Hungary.
3 Biomass Conversion Laboratory, Department of Energy, Tezpur University, Tezpur 784028, India.
* Correspondence: lonyi.ferenc@ttk.hu

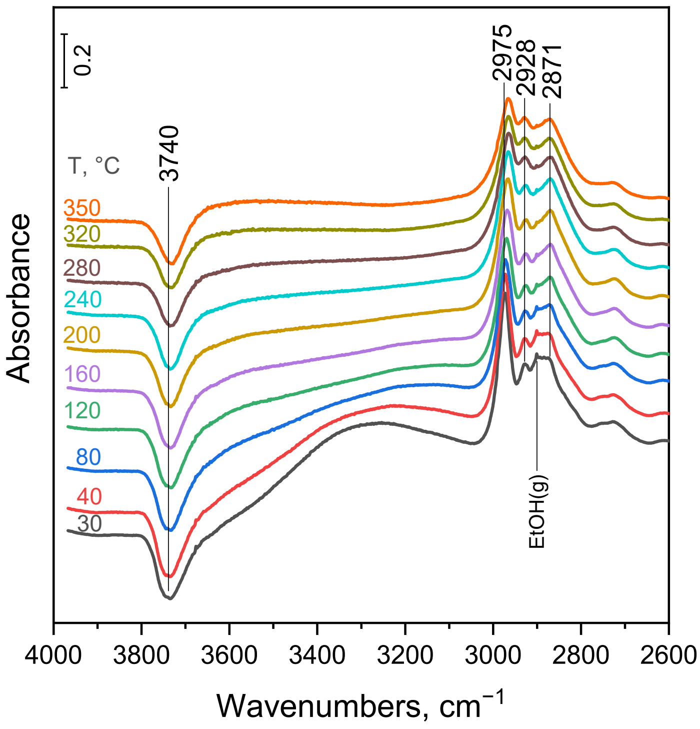

Figure S1. DRIFT spectra of the species obtained from adsorption and reaction of ethanol in He on MgO–Al_2O_3(550) catalyst in the frequency range of O–H and C–H stretching vibrations. The catalyst was contacted with a continuous flow of EtOH/He atmospheric gas mixture, while the temperature was continuously raised at a rate of 2 °C/min from 30 up to 350 °C. The corresponding spectrum of the neat catalyst was subtracted from the spectrum recorded at the indicated temperature.

The results obtained for the MgO–Al_2O_3(550) catalyst in contact with an EtOH/He reactant mixture are presented in Figure S1 (same as Figure 7A in the manuscript, except that the visible spectral range was extended to 4000 cm^{-1}). Adsorption of EtOH on the activated catalyst sample resulted in the appearance of C–H stretching bands due to the formation of surface

species, which was accompanied by the development of a negative absorption band at 3740 cm^{-1} in the range of the ν_{OH} stretching vibrations, suggesting that surface OH-groups are involved in adsorption interaction. The adsorbed species gave main characteristic absorption bands at 2975 and 2928 cm^{-1} in the frequency range of the ν_{CH} stretching vibrations, which can be assigned to the asymmetric stretching vibration of the –CH_3 (ν_{as}[CH_3]) and –CH_2– (ν_{as}[CH_2]) groups, respectively. A third band at 2871 cm^{-1} can be attributed to the symmetric stretching vibration of the –CH_3 groups (ν_s[CH_3]), probably overlapping with the band of symmetric –CH_2– vibration (a sharp band at 2901 cm^{-1} is also discernible due to the minor contribution of gas-phase ethanol).

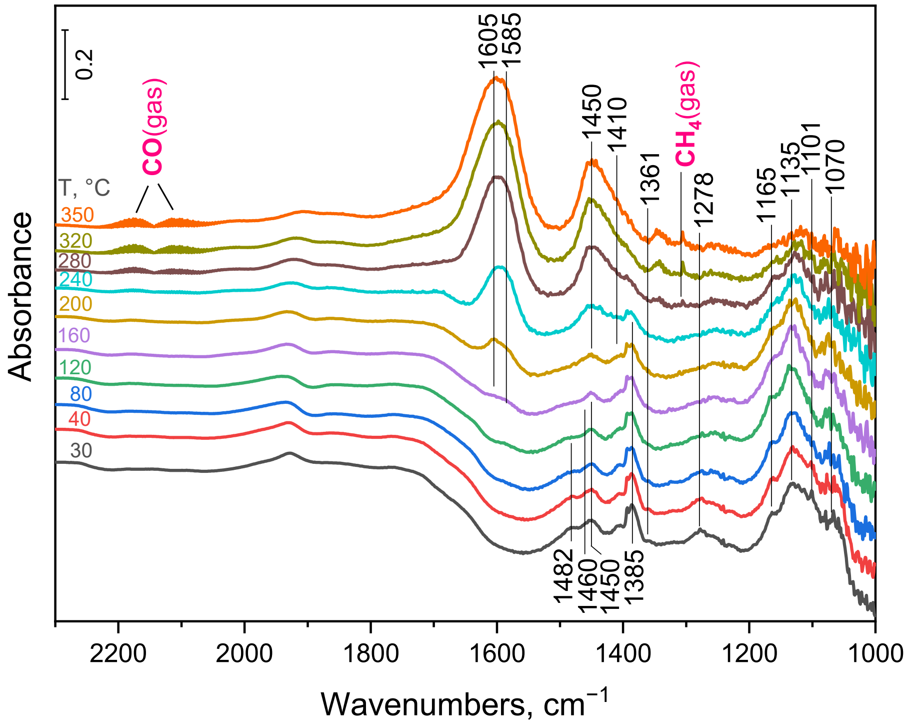

Figure S2. DRIFT spectra of the species obtained from adsorption and reaction of ethanol in H_2 on Pd/ MgO–Al_2O_3 catalyst in the frequency range of carbonyl stretching and C–H deformation vibrations. The catalyst was contacted with a continuous flow of EtOH/H_2 atmospheric gas mixture, while the temperature was continuously raised at a rate of 2 °C/min from 30 up to 350 °C. The corresponding spectrum of the neat catalyst was subtracted from the spectrum recorded at the indicated temperature.

The results obtained for the Pd/MgO–Al_2O_3 catalyst in contact with an EtOH/H_2 reactant mixture are presented in Figure S2 (same as Figure 10B in the manuscript, except that the visible spectral range was extended to 2300 cm^{-1}). Adsorption of EtOH on the activated catalyst sample resulted in the appearance absorption bands due to C–H deformation vibrations (1500–1000 cm^{-1}) and ν[O-C-O] stretching vibrations of carboxyl species (1700–1400 cm^{-1}) due to the formation of different surface species (for band assignments, see text in the manuscript). Results show that high reaction temperatures (≥280 °C) accelerated the

rate of the hydrodeoxygenation (HDO) reactions, as indicated by the bands of gas-phase CH_4 and CO.
